# Sexual health in older adults with hematologic malignancies – a sub-analysis of a Danish cross-sectional study

**DOI:** 10.1007/s00520-025-09921-9

**Published:** 2025-09-23

**Authors:** Melissa Street Chamberlin, Andrea Lange, Mary Jarden, Kristina Holmegaard Nørskov

**Affiliations:** 1 Department of Hematology, Copenhagen University Hospital Rigshospitalet, Copenhagen, Denmark; 2 Department of Oncology, Cancer Rehabilitation Research Center - Core Cancer, University Hospital Herlev and Gentofte, Herlev, Denmark; 3 Department of Clinical Medicine, University of Copenhagen, Copenhagen, Denmark; 4 Department of Hematology, Zealand University Hospital, Roskilde, Denmark

**Keywords:** Sexual health, Sexual dysfunction, Sexual symptoms, Hematologic malignancy, Older adults

## Abstract

**Purpose:**

This study aims to emphasize the importance of sexual health in older adults with hematologic malignancy (HM) in Denmark by exploring the prevalence of sexual symptoms and factors that influence sexual activity and function.

**Methods:**

A sub-analysis of a Danish cross-sectional study including older adults (≥ 65 years) diagnosed with a HM from 20/1–2013 to 20/8–2022. Eligible participants received electronic validated questionnaires about quality of life, sexuality, and sexual health in their officially assigned digital mailbox.

**Results:**

Decreased libido, impaired sexual activity, diminished confidence in erection, vaginal dryness, and reduced sexual satisfaction were among the most reported sexual symptoms. Interestingly, 93% of participants reported that sexual health had never been discussed with healthcare professionals. A significant correlation was found between sexual inactivity and a higher degree of sexual dysfunction in both men and women. Gender comparison revealed that men experienced significantly more sexual distress than women (*p* = 0.012). Age stratification showed that men aged 65–75 had significantly better erectile function compared to those > 75 (*p* = 0.031), while women aged 65–75 had better sexual arousal and orgasmic function than those > 75 years (*p* = 0.028).

**Conclusion:**

Older adults with HM experience a high prevalence of sexual dysfunction. Men > 75 years experienced more severe erectile dysfunction, and sexual distress was particularly notable among men and patients with lymphoma. Regardless of treatment, sexual dysfunction persists in older adults with HM, emphasizing the need for continuous, supportive care addressing sexual health both during and after treatment.

## Background

Sexuality is a multifaceted aspect of human life, influenced by biological, psychological and social factors [1, 2]. Life threatening diseases such as cancer can affect a person’s life in one or all three factors, making cancer a risk to sexual health [3]. While research in sexual health and cancer has primarily focused on gender specific cancers, the impact on patients with hematological malignancies (HM) remains largely overlooked [2, 4]. A systematic review of oncology studies from 2008 to 2014 found that only 11% included sexual function as a patient-reported outcome, highlighting a significant underrepresentation [5]. Current research tend to focus on younger populations and the sexual symptom burden in a short-term perspective, whereas research regarding the older HM population is sparse [2, 4].

Older adults are particularly vulnerable to changes in sexual health. Sexual symptoms naturally increase with age due to physiological changes and lifestyle-related conditions [3, 6]. In menopausal women, changes in hormone levels can lead to vaginal wall atrophy, reduced lubrication, and vaginal dryness, which often result in dyspareunia [7]. In men, declining testosterone levels can result in decreased sexual desire, erectile dysfunction (ED), diminished orgasm and longer refractory periods [7].

The incidence of HM is increasing due to a growing older adult population (age ≥ 65 years) [8], improved diagnostics, and new treatment strategies [9]. Although survival rates have improved, treatments often come with a significant symptom burden, including sexual symptoms [9–11]. Specifically, treatment for HM can exacerbate existing sexual symptoms in older adult patients. These factors can have a detrimental effect on sexual health, including sexual activity and function [3, 6].

Sexual symptoms are a common late sequelae in cancer survivors with a prevalence of 40–100% [12]. Frequent sexual symptoms reported by men include ED and decreased satisfaction with sexual activity and frequency [6, 13]. Women most commonly report vaginal dryness and dyspareunia [3, 13]. Both genders experience imbalance in sex hormones and reduced libido [13]. These sexual symptoms may be temporary or long term [13]. Despite the negative impact of these symptoms on sexual health and quality of life (QoL), they are often an underrecognized side effect of HM [9, 14].

In Western European society, ageism is prevalent and often contributes to the stereotype of older adults as asexual [15]. However, studies have shown that older adults remain sexually active and view sexuality as an important aspect of QoL and well-being [7, 16, 17]. Ageism fosters a two-way taboo, where both the healthcare professional (HCPs) and patients avoid discussing sexual concerns [18–20]. This stigma leads to the neglect of sexual health issues among older adults [15].

This study aims to emphasize the importance of sexual health in older adults with HM in Denmark by exploring the prevalence of sexual symptoms and the factors that influence sexual activity and function.

## Material and methods

This study is a sub analysis of a cross-sectional study conducted at the Department of Hematology, Copenhagen University Hospital, Rigshospitalet, Denmark. The study was carried out in accordance with the STROBE guideline for reporting observational studies [21]. Further details regarding the primary study has been published elsewhere [22].

### Participants and recruitment

All participants ≥ 65 years and diagnosed with one of the following HM between January 20, 2013 and August 20, 2022, were included: acute myeloid/lymphatic leukemia (AL), chronic myeloid/lymphatic leukemia (CL), multiple myeloma (MM), lymphoma, or myelodysplastic syndrome (MDS).

Participants were identified through the National Patient Register, from which a representative sample was extracted by the Danish Health Data Authority on January 1, 2023 [22].

### Data collection

Medical and sociodemographic data were collected directly from the participants. Patient-reported outcomes included the following validated questionnaires; The European Organization for Research and Treatment of Cancer Quality of Life Questionnaire – Sexual Health (EORTC QLQ-SH22), The European Organization for Research and Treatment of Cancer Quality of Life Questionaire-C30 (EORTC QLQ-C30), International Index of Erectile Function (IIEF-15), Female Sexual Function Index (FSFI) and Female Sexual Distress Scale-Revised (FSDS-R).

#### Primary outcome

The primary outcome was sexual function and was assessed using validated, gender specific instruments. For men, the IIEF evaluated erectile function in five domains; erectile function, orgasmic function, sexual desire, intercourse satisfaction, and overall satisfaction. Higher scores indicates better functioning, whereas an erectile function score below 26.0 indicates ED [23, 24]. For women, the FSFI was used to evaluate sexual function in six domains; desire, arousal, lubrication, orgasm, satisfaction, and pain, with higher scores indicating better sexual function. A cut-off score of 26.55 indicates sexual dysfunction [25].

#### Secondary outcome

QoL was assessed using the EORTC QLQ-C30, which measures QoL across three domains; global QoL, function and symptoms, with scores ranging from 0–100. Higher scores on the global QoL and functional scales indicate better QoL and function, while higher scores on the symptom scale indicate greater symptom burden [26, 27]. Sexual health was assessed using the EORTC QLQ-SH22, where higher scores (0–100) indicate a higher symptom burden [28]. Sexual distress was evaluated using the FSDS-R for both men and women, with a cut-off score ≥ 11 indicating sexual distress [29].

### Ethics

Before participating in the study, all participants were provided with written information about the study, including details on ethical considerations such as data anonymization and voluntary participation. By completing the questionnaires, participants provided their informed consent. Given the sensitive nature of the research, participants were informed that they could contact the primary investigator if they experienced any unexpected concerns during their participation in the study.

### Statistical analysis

Sample sizes in the original study were calculated to allow comparison of diagnosis groups for each gender-specific primary outcome [22]. In total, 201 of the 362 participants from the original study were included in the sub-analysis. For the FSFI sum score we observed a standard deviation of approximately 10 in contrast to the value 6.75 used for the sample size calculation. This implies that the study may be slightly underpowered for women. Conversely, for the IIEF EF-score, the standard deviation was below the value 10 used for the sample size calculation, except in patients with AL and lymphoma.

Official scoring manuals, including guidelines for handling missing responses, were used for computation of subscale scores for questionnaires. Descriptive statistics were used to calculate means and percentages for each subscale. Data were presented as mean, standard deviation (SD) and *p*-value. Group comparisons across diagnosis were assessed using ANOVA. Sociodemographic and clinical data were summarized as numbers and percentages. To analyze differences in sexual function and sexual health, mean scores were compared across categorical variables using independent *t*-test: gender, age (65–75 years/> 75 years), marital status (cohabiting/single), treatment status (yes/no) and sexual activity (active/inactive). We categorized participants into age groups of 65–75 ("young-old") and > 75 ("old-old") based on well-established distinctions in gerontological and clinical literature [30, 31]. This binary reflects meaningful differences in health status, functional capacity, and aging trajectories. Prior studies have employed similar cut-offs, supporting the relevance and comparability of our approach [17]. The independent *t*-test was considered most suitable given the small sample size in the subgroups. Univariate analyses were performed to examine the associations between individual sociodemographic and clinical variables and the likelihood of experiencing sexual dysfunction among men and women. Statistical significance was set at a 95% confidence interval (CI), with a two-sided *p*-value ≤ 0.05. All analysis were performed using IBM SPSS version 29.0.1.0.

## Results

A total of 201 participants were included in this sub-analysis, with a mean age of 74.3 years (range: 65–91) and 53.2% were men. The majority were married or cohabiting (70.1%) and retired (89.6%). Diagnoses were distributed as follows: CL (28.9%), MM (22.9%), MDS (13.9%), AL (12.4%), and lymphoma (11.9%). Participants had been diagnosed from less than one year to over seven years ago, with most still in follow-up. Treatment status varied across diagnostic groups (Table 1), reflecting underlying differences in disease characteristics. Patients with lymphoma, CL, and AL were more likely to be in follow-up care, whereas among patients with MDS and multiple myeloma, treatment status was more evenly distributed between active treatment and follow-up.

**Table 1:** Demographic and clinical characteristics

Characteristic	Total Sample	Acute Leukemia	Chronic leukemia	Lymphoma	Myelodysplastic syndrome	Multiple myeloma
	(*N* = 201)	(*N* = 25)	(*N* = 59)	(*N* = 24)	(*N* = 27)	(*N* = 46)
Gender (female), n (%)	94 (46.8)	10 (40)	30 (50.8)	14 (58.3)	11 (40.7)	20 (43.5)
Gender (men), n (%)	107 (53.2)	15 (60)	29 (49.2)	10 (41.7)	16 (59.3)	26 (56.5)
Age, mean (SD)	74.3 (6.1)	73.3 (3.8)	74.8 (5.8)	73.9 (6.4)	74.5 (6.8)	73.8 (6.3)
**Education, n (%)**						
No high school degree	24 (11.9)	5 (20)	4 (6.8)	2 (8.3)	2 (7.4)	7 (15.2)
Vocational education	56 (27.9)	9 (36)	20 (33.9)	3 (12.5)	9 (33.3)	8 (17.4)
High school degree	1 (0.5)	0	0	0	0	0
2 year college	26 (12.9)	4 (16)	6 (10.2)	3 (12.5)	4 (14.8)	6 (13)
4 year college	61 (30.3)	5 (20)	17 (28.8)	12 (50)	9 (33.3)	15 (32.6)
Master´s degree or higher	33 (16.4)	2 (8)	12 (20.3)	4 (16.7)	3 (11.1)	10 (21.7)
Unknown	3 (0.9)	0	1 (1.1)	0	0	1 (1.5)
**Marital status, n (%)**						
Married or cohabitating	141 (70.1)	15 (60)	41 (69.5)	17 (70.8)	21 (77.8)	36 (78.3)
Single, separated, divorced, or widowed	56 (27.9)	8 (32)	17 (28.8)	7 (29.2)	6 (22.2)	9 (19.6)
Unknown	4 (2)	2 (8)	1 (1.7)	0	0	1 (2.2)
**Occupation, n (%)**						
Salaried employee	16 (8)	3 (12)	4 (6.8)	0	2 (7.4)	5 (10.9)
Unemployed	4 (2)	2 (8)	0	1 (4.2)	1 (3.7)	0
Retired employee	180 (89.6)	20 (80)	54 (91.5)	23 (95.8)	24 (88.9)	41 (89.1)
Unknown	1 (0.5)	0	1 (1.7)	0	0	0
**Time since diagnosis, n (%)**						
< 1 year	7 (3.5)	1 (4)	2 (3.4)	0	1 (3.7)	3 (6.5)
1—3 years	64 (31.8)	7 (28)	16 (27.1)	11 (45.8)	7 (25.9)	14 (30.4)
4—7 years	43 (21.4)	4 (16)	18 (30.5)	0	6 (22.2)	11 (23.9)
> 7 years	75 (37.3)	9 (36)	19 (32.2)	13 (54.2)	10 (37)	17 (37)
Unknown	12 (6)	4 (16)	4 (6.8)	0	3 (11.1)	1 (2.2)
**HSCT, donor source, n (%)**						
Autologous	26 (12.9)	1 (4)	0	3 (12.5)	0	22 (47.8)
Allogeneic	19 (9.5)	11 (44)	1 (1.7)	0	5 (18.5)	2 (4.3)
**Treatment status**						
In treatment	58 (28.9)	6 (24)	9 (15.3)	2 (8.3)	11 (40.7)	25 (54.3)
In follow-up	118 (58.7)	13 (52)	43 (72.9)	16 (66.7)	14 (51.9)	19 (41.3)
End of treatment	21 (10.4)	6 (24)	5 (8.5)	6 (25)	2 (7.4)	1 (2.2)
Unknown	1 (0.5)	0	0	0	1 (3.7)	0

*SD* standard deviation, *HSCT* Hematopoetic stem cell transplantation

### Sexual health and symptoms

We studied multiple sexual health domains (Table 2). The most affected sexual symptoms were reduced sexual activity due to treatment (42.6 ± 40.4), sexual satisfaction (48.9 ± 25.6), decreased libido (51.4 ± 37.8), vaginal dryness (48.1 ± 33.3), and confidence in erection (55.9 ± 38.2). In total 93% of participants had not discussed sexual issues with a HCP, which was consistent across diagnoses.

**Table 2:** Patient-reported outcomes across diagnoses

Variables	All participants		Acute leukemia		Chronic leukemia		Lymphoma		Myelodysplastic syndrome		Multiple myeloma	
	(*N* = 201)		(*N* = 25)		(*N* = 59)		(*N* = 24)		(*N* = 27)		(*N* = 46)	
	*N*	Mean (SD)	*N*	Mean (SD)	*N*	Mean (SD)	*N*	Mean (SD)	*N*	Mean (SD)	*N*	Mean (SD)
**EORTC QLQ-C30**												
Global Health (0–100)	*200*	69.4 (21.8)	*25*	66.3 (21.7)	*59*	73.2 (25.3)	*24*	60.8 (16.8)	*27*	68.8 (21.3)	45	70.9 (17.8)
Physical functioning (0–100)	*200*	78.5 (20.6)	*25*	79.5 (16.8)	*59*	82.9 (21.5)	*24*	70.3 (22.4)	*27*	79 (21.3)	45	77.3 (19.8)
Role functioning (0–100)	*200*	77.1 (26.8)	*25*	75.3 (23.1)	*59*	84.7 (25.4)	*24*	69.4 (28.5)	*27*	76.5 (23.7)	45	73.7 (28.1)
Emotional functioning (0–100)	*200*	80.6 (22.8)	*25*	86 (21.5)	*59*	82.5 (24.4)	*24*	75.7 (26)	*27*	77.2 (19)	45	83.1 (18.1)
Cognitive functioning (0–100)	*200*	82.9 (20.5)	*25*	86 (19.1)	*59*	85.6 (19.9)	*24*	76.4 (27.3)	*27*	82.1 (19.6)	45	84.1 (15.5)
Social functioning (0–100)	*200*	83.1 (23.1)	*25*	82 (19.8)	*59*	88.1 (21.2)	*24*	73.6 (28.2)	*27*	79.6 (22.3)	45	85.6 (18.7)
Fatigue (0–100)	*200*	34.3 (26.4)	*25*	32.9 (24.3)	*59*	28.6 (30.5)	*24*	44.4 (24.3)	*27*	38.7 (22.7)	45	31.6 (21.6)
Nausea and vomiting (0–100)	*200*	7.8 (17)	*25*	9.3 (16)	*59*	7.1 (17.8)	*24*	7.6 (13.9)	*27*	6.8 (13.3)	45	6.3 (18.2)
Pain (0–100)	*200*	22.2 (27.8)	*25*	19.3 (23.9)	*59*	17.8 (30.3)	*24*	36.8 (28.6)	*27*	22.8 (25.4)	45	21.9 (23.8)
Dyspnoea (0–100)	*200*	18.5 (24.9)	*25*	20 (25.5)	*59*	14.7 (22.5)	*24*	29.2 (26.6)	*27*	13.6 (16.7)	45	14.1 (23)
Insomnia (0–100)	*200*	27.2 (31)	*25*	18.7 (27.4)	*59*	26 (33.9)	*24*	29.2 (28.3)	*27*	35.8 (31.9)	45	23.7 (25.2)
Appetite loss (0–100)	*199*	13.7 (26.2)	*25*	13.3 (21.5)	*59*	12.4 (29.6)	*23*	13 (19.4)	*27*	12.3 (24.7)	45	10.4 (24.4)
Constipation (0–100)	*200*	15.5 (24.5)	*25*	12 (23.3)	*59*	19.2 (25.7)	*24*	13.9 (19.5)	*27*	11.1 (22.6)	45	15.6 (24.2)
Diarrhoea (0–100)	*198*	13.1 (23.2)	*25*	6.7 (16.7)	*59*	14.1 (24.9)	*22*	9.1 (15.2)	*27*	12.3 (21)	45	15.6 (23.1)
Financial difficulties (0–100)	*198*	5.9 (16.2)	*25*	5.3 (15.8)	*57*	4.7 (16)	*24*	11.1 (21.2)	*27*	4.9 (17.8)	45	5.9 (14.7)
**EORTC QLQ-SH22**												
Sexual activity (0–100)	*189*	62.3 (33.7)	*24*	59.7 (35.4)	*55*	57 (35.5)	23	60.9 (34.3)	25	66.7 (27.2)	43	69 (28.5)
Sexual satisfaction (0–100)	*149*	48.9 (25.6)	*21*	38.6 (27.2)	*44*	48.3 (26.1)	15	55.8 (25.1)	22	54.6 (27.5)	33	49.9 (18.1)
Sexual pain (0–100)	*146*	9.2 (21.4)	*21*	14 (31.3)	*40*	6.8 (18)	17	23.9 (31.8)	21	5.6 (16.1)	33	4.5 (12.1)
Decreased Libido (0–100)	*186*	51.4 (37.8)	*23*	44.9 (35.7)	*55*	49.1 (40)	22	53 (40.7)	27	63 (33.8)	41	52.8 (36.5)
Incontinence (0–100)	*183*	21.9 (33.5)	*23*	15.9 (28.2)	*52*	26.3 (36.4)	22	21.2 (26.3)	26	15.4 (31.6)	42	20.6 (31.2)
Fatigue (0–100)	*186*	32.4 (35.8)	*23*	33.3 (37.6)	*54*	32.7 (37.5)	22	33.3 (35.6)	27	28.4 (34.2)	42	33.3 (33.7)
Treatment (0–100)	*151*	42.6 (40.4)	*23*	50.7 (41.3)	*40*	31.7 (40.6)	18	57.4 (42.5)	23	34.8 (42)	33	50.5 (36.4)
Communication with professionals (0–100)	*187*	93.4 (16.9)	*23*	94.2 (12.9)	*55*	93.9 (18.2)	23	87 (26.1)	27	96.3 (14.1)	41	92.7 (14)
Partnership (0–100)	*129*	38.5 (38.5)	*17*	27.5 (39.5)	*38*	43.9 (42.5)	16	45.8 (36.3)	19	38.6 (37.3)	26	42.3 (36)
Confidence Erection (0–100)	*102*	55.9 (38.2)	*15*	51.1 (35.3)	*28*	59.5 (40.9)	10	56.7 (35.3)	16	60.4 (40.8)	23	58 (35.1)
Body image (men) (0–100)	*103*	25.9 (32.3)	*15*	22.2 (27.2)	*28*	27.4 (32.8)	10	46.7 (47.7)	16	20.8 (26.9)	24	26.4 (29.5)
Body image (women) (0–100)	*87*	18 (29.6)	*8*	20.8 (30.5)	*29*	16.1 (26.2)	13	20.5 (37.4)	11	36.4 (34.8)	18	3.7 (10.8)
Vaginal dryness (0–100)	*36*	48.1 (33.3)	*5*	46.7 (29.8)	*12*	52.8 (36.1)	6	66.7 (42.2)	3	44.4 (38.5)	6	27.8 (13.6)
**FSDS-R**												
Global score (0–52)	*172*	11.8 (13.1)	*23*	10.5 (12.2)	*50*	13.5 (14.9)	22	14.4 (14.7)	24	11.3 (11.8)	37	9.9 (10.1)
**FSFI (women)**												
Sum score (2–36)	*56*	13.9 (10.3)	*6*	23.4 (10.2)	*19*	13.6 (11.4)	*7*	10.8 (3.5)	8	9.2 (7.7)	9	17.1 (11.4)
Desire (1.2–6)	*80*	2.2 (1.1)	*8*	2.7 (1.4)	*27*	2.3 (1.1)	*12*	2.5 (1.1)	11	1.7 (0.8)	15	2 (1.1)
Arousal (0–6)	*80*	1.8 (1.9)	*8*	2.9 (2.4)	*27*	1.9 (2.1)	*12*	1.4 (1.4)	11	1.4 (1.5)	15	1.9 (2.1)
Lubrication (0–6)	*77*	1.5 (1.9)	*8*	2.7 (2.3)	*26*	1.6 (2.2)	*11*	1 (1.4)	11	1 (1.7)	14	1.5 (1.9)
Orgasm (0–6)	*76*	1.9 (2.4)	*8*	3.9 (2.7)	*26*	1.7 (2.5)	*10*	1.5 (2)	11	1.6 (2.4)	14	2 (2.6)
Satisfaction (0.8–6)	*58*	3.6 (1.6)	*6*	4.6 (1.5)	*19*	3.4 (1.8)	*8*	2.8 (1.3)	8	3 (2)	10	4.4 (1)
Pain (0–6)	*74*	1.6 (2.4)	*7*	3.1 (3)	*25*	2 (2.7)	*10*	0.6 (1.1)	11	0 (0)	14	1.9 (2.7)
**IIEF-15 (Men)**												
Sum score	*89*	28.4 (20.8)	*13*	33.7 (22.7)	*22*	24.1 (19.4)	10	33 (22.4)	14	19.4 (17.1)	22	28.9 (20.2)
Erectile Function (0–30)	*96*	9.5 (9.3)	*14*	11.8 (10.5)	*26*	7.7 (8.1)	10	12.3 (9.7)	14	5.8 (7.4)	23	9.5 (9.6)
Orgasmic Function (0–10)	*99*	4.3 (4.5)	*15*	4.9 (4.4)	*26*	3 (4.1)	10	4.8 (4.4)	14	2.9 (4.7)	24	5.6 (4.6)
Sexual Desire (1–10)	*98*	5 (2.4)	*15*	4.8 (2.3)	*27*	5.4 (2.5)	10	5.5 (2.7)	14	3.9 (2.2)	22	5 (2.4)
Intercourse Satisfaction (0–15)	*97*	3.6 (4.7)	*14*	5.9 (5)	*26*	2.5 (4.1)	10	4.7 (5.2)	14	1.6 (3.8)	24	3.4 (4.3)
Overall Satisfaction (1–10)	*96*	5.5 (2.5)	*15*	5.7 (2.3)	*24*	5.1 (3)	10	5.7 (2.2)	14	5.1 (2.9)	24	5.4 (2)

*EORTC QLQ-C30* The European organization for research and treatment of cancer quality of life questionnaire, *EORTC QLQ-SH22* The european organization for research and treatment of cancer quality of life questionnaire sexual health, *FSDS-R* Female sexual distress scale – revised, *FSFI* Female sexual function index, *IIEF-15* The international index of erectile function questionnaire, *SD* standard deviation

In men, the mean EF-score across all diagnoses was 9.5 ± 9.3, with 91.7% below cut-off for ED. The mean EF-score was significantly lower in men aged > 75 years compared to those aged 65–75 years (6.2 ± 7.9 vs. 10.1 ± 9.8; *p* = 0.031), see Table 3. Men undergoing active treatment had lower EF-scores compared to those in inactive treatment (6.5 ± 7.7 vs. 9.3 ± 9.7; *p* = 0.114). A subgroup analysis showed that treatment had a significantly greater impact on sexual activity in men compared to woman (50.2 ± 39.9 vs. 32.3 ± 38.9; *p* = 0.007). Univariate analyses revealed that age was significantly associated with increased odds of sexual dysfunction, with older men being more likely to report dysfunction (OR:1.20; 95%CI [1.03–1.47]; *p* = 0.037). In contrast, higher levels of physical functioning were associated with a reduced likelihood of experiencing sexual dysfunction (OR: 0.88; 95%CI [0.75–0.97]; *p* = 0.049).

**Table 3:** Sexual function in age groups

		65–75 years (*n* = 113)		> 75 years (*n* = 78)	
Variables	N	Mean (SD)	N	Mean (SD)	*P*-value [95%CI]
**FSFI (women)**	51	2.3 (1.1)	29	2.0 (1.1)	0.259 [−0.2; 0.8]
Sum score (2–36)	*51*	2.2 (1.9)	29	1.2 (1.6)	0.028 [0.1; 1.8]
Desire (1.2–6)	*49*	1.8 (2.0)	28	0.9 (1.7)	0.064 [−0.1; 1.8]
Arousal (0–6)	*48*	2.3 (2.5)	28	1.1 (2.1)	0.028 [0.1; 2.4]
Lubrication (0–6)	*38*	3.5 (1.7)	20	3.6 (1.5)	0.786 [−1.0; 0.8]
Orgasm (0–6)	*48*	1.9 (2.6)	26	1.0 (2.1)	0.130 [−0.3; 2.1]
Satisfaction (0.8–6)	*37*	15.3 (10.4)	19	11.2 (9.9)	0.155 [−1.6; 9.9]
Pain (0–6)					
**IIEF-15 (Men)**					
Sum score	*55*	32.4 (21.6)	33	22.1 (18.1)	0.024 [1.4; 19.2]
Erectile Function (0–30)	*56*	11.5 (9.7)	*38*	6.2 (7.9)	0.023 [0.6; 8.2]
Orgasmic Function (0–10)	*57*	5.3 (4.6)	*39*	2.9 (3.9)	0.008 [0.6; 4.2]
Sexual Desire (1–10)	*57*	5.2 (2.5)	*39*	4.5 (2.4)	0.746 [−0.6; 1.4]
Intercourse Satisfaction (0–15)	*56*	4.3 (5.1)	*39*	2.4 (3.7)	0.041 [0.1; 3.9]
Overall Satisfaction (1–10)	*57*	6.0 (2.5)	*36*	4.7 (2.4)	0.015 [0.3; 2.3]

*FSFI* Female sexual function index, *IIEF-15* The international index of erectile function questionnaire, *SD* standard deviation

In women, the mean FSFI sum score across diagnosis was 13.9 ± 10.3, with 80.4% scoring below the cut-off indicating a high prevalence of sexual dysfunction. Sexual function was notably impacted in domains related to arousal, lubrication, orgasm, and dyspareunia. Women aged 65–75 years reported significantly better sexual arousal (*p* = 0.028) and better orgasmic function (*p* = 0.028) than women aged > 75 years (Table 3). Sexual dysfunction was prevalent regardless of treatment stage. However, women in follow-up or post-treatment phases had lower, though not statistically significant, FSFI scores compared to those currently undergoing treatment (13.0 ± 10.1 vs. 16.5 ± 10.8; *p* = 0.277).

Univariate analyses revealed that several variables were significantly associated with sexual dysfunction in women. Specifically, lower levels of physical functioning were linked to increased odds of sexual dysfunction (OR: 0.89; 95%CI [0.81–0.95]; *p* = 0.006), as were reduced role functioning (OR: 0.89; 95%CI [0.76–0.95];* p* = 0.025), and poorer cognitive functioning (OR: 0.97; 95%CI [0.73–0.95]; *p* = 0.023). In addition, higher levels of fatigue (OR: 1.10; 95%CI [1.05–1.21]; *p* = 0.001) and pain (OR: 1.07; 95%CI [1.02–1.12]; *p* = 0.028) were significantly associated with greater odds of sexual dysfunction.

### Sexual activity

Overall, 51.9% of participants were sexually active, with a higher percentage of men (62.2%). Sexual function was significantly worse in sexually inactive men (2.5 ± 2.7 vs. 13.3 ± 9.6; *p* < 0.001) and women (6.4 ± 4.0 vs. 21.4 ± 9.4; *p* < 0.001). Sexually active participants were significantly more satisfied with their partnership and sexual life (Table 4).

**Table 4:** Sexual activity across global and sexual health domains

		Sexual active		Sexual inactive	
	N	Mean (SD)	N	Mean (SD)	*P*-value [95%CI]
**EORTC QLQ-C30**					
Global Health (0–100)	98	75.1 (19.7)	91	63.4 (22.4)	< 0.001 [−17.8; −5.7]
**EORTC QLQ-SH22**					
Communication with professionals (0–100)	98	94.7 (16.7)	88	92.2 (17.1)	0.312 [−2.4; 7.4]
Partnership (0–100)	85	32.9 (35.8)	43	49.6 (42.0)	0.021 [2.6; 30.7]
Body image (men) (0–100)	61	25.1 (32.0)	42	26.9 (33.1)	0.777 [−11.1; 14.8]
Body image (women) (0–100)	37	12.6 (25.3)	49	22.4 (32.2)	0.129 [−2.9; 22.6]
Sexual satisfaction (0–100)	98	38.5 (29.9)	51	68.7 (19.9)	< 0.001 [22.9; 37.4]
**IIEF-15 (Men)**					
Sum score	55	38.9 (19.6)	34	11.3 (6.5)	< 0.001 [−34.6; −20.8]
Erectile Function (0–30)	61	13.3 (9.6)	42	2.5 (2.7)	< 0.001 [−13.9; −7.8]
**FSFI (women)**					
FSFI sum score	27	21.4 (9.4)	28	6.4 (4.0)	< 0.001 [−18.9; −11.1]
**FSDS-R**					
Global score (0–52)	89	11.9 (11.7)	82	11.8 (14.5)	0.922 [−4.2; 3.8]

*EORTC QLQ-C30* The European organization for research and treatment of cancer quality of life questionnaire, *EORTC QLQ-SH22* The European organization for research and treatment of cancer quality of life questionnaire sexual health, *FSDS-R* Female sexual distress scale – revised, *FSFI* Female sexual function index, *IIEF-15* The International index of erectile function questionnaire, *SD* standard deviation

### Sexual distress

Sexual distress was observed in most diagnosis groups, with a mean FSDS-R score of 11.3 ± 13.1 except for AL (10.5 ± 12.2) and MM (9.9 ± 10.1) (Table 2). Patients with lymphoma reported the highest level of sexual distress (14.4 ± 14.7).

Men reported significantly more sexual distress than women (14.1 ± 13.6 vs. 11.9 ± 12.0; *p* = 0.012). However, among participants above the cut-off for sexual distress (*n* = 72), no significant difference between genders was observed (*p* = 0.297). Additionally, no difference in sexual distress was found between sexually active (11.9 ± 11.7) and sexually inactive participants (11.8 ± 14.5; *p* > 0.05).

### Quality of life

The mean global QoL score for all diagnoses was 69.4 ± 21.8. A positive association was found between global QoL and sexual activity, with sexually active participants reporting significantly higher global QoL scores (75.1 ± 19.7 vs. 63.4 ± 22.4; *p* < 0.001) (Table 4). Lastly, QoL was significantly associated with sexual function. Among women, lower overall QoL scores were linked to an increased likelihood of sexual dysfunction (OR: 0.916; 95%CI [0.86–0.96]: *p* = 0.003). In men, linear regression analysis indicated that sexual dysfunction was significantly associated with lower global QoL scores (β = 0.119; *p* = 0.005).

## Discussion

This sub-analysis examined the sexual health of older patients with HM in Denmark and explored factors associated with sexual activity and functioning. The findings provide valuable insights into potential areas for targeted interventions both during and after treatment. We identified a high prevalence of sexual dysfunction affecting patients up to 10 years post diagnosis, irrespective of treatment trajectory and time since diagnosis. Gender comparisons revealed that men experienced higher levels of sexual distress, with those above 75 years reporting more severe ED.

Sexual dysfunction often increases with age, as the sexual response cycle can be affected by age-related changes [17, 32]. Our study revealed a high prevalence of sexual dysfunction, with decreased sexual activity and function among older adults with HM. Findings from an integrative review shows that sexual function in older cancer survivors is significantly decreased 5–10 years post treatment, regardless of cancer type [33]. The prevalence and the severity of dysfunction were notably higher in our study compared to previous studies in younger populations, suggesting that sexual dysfunction is more pronounced in older adults with HM [3, 6]. To contextualize this finding, a Danish population-based investigation of sexual health among a nationally representative sample (*n* = 62,675) found that among respondents aged 65–74, 21% of men and 35% of women reported sexual difficulties such as reduced desire, arousal challenges, or pain during sexual activity. Yet fewer than 10% had ever discussed these issues with a healthcare professional [17]. In comparison, sexual dysfunction appears both more prevalent and more clinically neglected in our sample of older adults with HM. This suggests additive or compounding effects of cancer, its treatment, and aging on sexual health in this population. Notably, these patterns emerge within the Danish context, where public healthcare access and cultural openness regarding sexuality might be expected to facilitate greater attention to sexual well-being. Despite the high prevalence of sexual dysfunction, a substantial proportion of older adults remained sexually active. Given that only a small proportion of participants had discussed sexual concerns with a healthcare professional may help explain the high levels of dysfunction observed in our study. Prior research has shown that patients who engage in conversations about sexual health with clinicians tend to report greater sexual satisfaction and reduced anxiety related to sexual activity [34]. This underscores the potential benefit of proactively offering discussions about sexuality as part of follow-up care.

In our study, men reported a high prevalence of severe ED. In the general population, ED and hypogonadism are the leading causes of sexual dysfunction in men, with the prevalence increasing with age [7, 17]. As men age, natural declines in testosterone levels contribute to reduced libido. Sexual arousal becomes slower, the time required to reach orgasm lengthens, and erections require more physical stimulation, becoming less frequent and less durable [7]. Despite these age-related changes, men in our study reported lower levels of sexual activity and more severe sexual function compared to younger patients with HM, patients with other cancer types, and the general population [17, 22, 35–37]. This suggest that older men with HM are particularly vulnerable to sexual health problems.

We found that women reported poorer sexual function compared to younger women with HM, other cancers, and the general population [3, 17, 38]. Specifically, a systematic review and meta-analysis of women with urogenital, mammae, and colorectal cancer found a prevalence of sexual dysfunction of 60% [38]. In contrast, women in our study reported a prevalence of sexual dysfunction of 80%. This difference could be attributed to age and cancer treatment regimen. Increasing age is negatively correlated with sexual dysfunction in patients with HM [3, 6]. In addition, studies have found that sexual dysfunction is more prevalent in patients treated with chemo- and radiotherapy compared to surgery [39, 40]. A variety of side effects to chemotherapy can negatively affect sexuality, e.g. vaginal dryness, hormonal imbalance, fatigue, dyspareunia, and distress [3, 6, 13, 41, 42]. Further, women can experience age related sexual problems, including vaginal atrophy, reduced vaginal lubrication, and decreased sensitivity in erogenous zones affecting libido [7, 32]. Consequently, the risk of sexual dysfunction may be further increased in older women following the diagnosis and treatment for HM. Partnered or marital status is another important factor when investigating sexual function, particularly in relation to gender differences. While our main study acknowledged that the scoring of both the FSFI and IIEF can be lowered by the absence of sexual activity we therefore conducted subgroup analyses limited to sexually active participants to better isolate dysfunction. Still partnered status may shape the opportunity for sexual engagement, particularly among older adults [22]. Women are more likely to be unpartnered in later life, and among those not sexually active, low FSFI scores may reflect lack of opportunity rather than true sexual dysfunction [43]. This suggests that some of the observed gender differences in sexual function scores may be influenced more by social and relational factors than by physiological dysfunction alone and should therefore be interpreted with caution. As many remain sexually active despite their age and sexual dysfunctions, highlights the importance of being proactive in initiating conversations about sexual health during their treatment, regardless of age.

Sexual distress was found to be higher among men than women. In addition, we found a high prevalence of ED with the highest severity among men aged over 75 years. A plausible explanation for the gender differences in sexual distress could be differences in age-related physiological variations. The andropause and the associated clinical symptoms of testosterone decline typically emerge around the age of 75, whereas menopause generally begins earlier, in midlife [7]. As men transition into andropause around age 75, women may have already adapted to the physiological changes associated with menopause, which could explain why women may be more accepting of their sexual challenges. Sexual distress levels decrease in aging women despite increasing sexual dysfunction, is a tendency, that has been observed in the general Danish population [17].

We identified the poorest QoL-score among patients with lymphoma. Simultaneously, this group demonstrated the lowest levels of sexual activity, reported greater sexual symptoms and side effects, and exhibited the highest levels of sexual distress. Importantly, reviews have highlighted that QoL in patients with lymphoma is often compromised due to late sequelae and fear of recurrence [42, 44, 45], with psychological distress correlating with reduced sexual activity among male lymphoma survivors [42]. These findings suggest that older patients with lymphoma are more vulnerable to sexual health problems and might, in particular, have a greater need for sexual rehabilitation and supportive care.

Studies among older adults have demonstrated that sexuality is an important part of a healthy relationship and overall well-being [7, 16]. Simultaneously, problems related to sexuality and sexual function are known to negatively affect QoL [17]. However, limited research has investigated the association between QoL and sexual symptoms among patients with HM [2, 10]. Olsson et al. found that fewer sexual symptoms were associated with better QoL six months after chemotherapy and immunotherapy for patients with lymphoma and leukemia [10]. However, these findings contradict a longitudinal study by the same authors, which found increased sexual symptoms and better QoL in patients with lymphoma and leukemia one month after chemotherapy or immunotherapy [2]. Since a cancer diagnosis can be distressing and may overshadow concerns related to sexual health [4, 46, 47], these contradicting results may be explained by the timing of measurement of sexual symptoms. Nevertheless, an integrative review of sexual health among older cancer survivors finds that sexual health related QoL is negatively affected by cancer diagnosis and treatment regardless of cancer type [33]. If sexual problems persist for years after the initial diagnosis in older survivors of HM, these findings suggest that long-term survivors may face ongoing challenges that adversely affect their overall QoL. This is an important finding, as research indicate that sexual health plays a significant role in influencing QoL, with a positive sexual well-being being associated with reduced emotional distress, improved mental health, and better coping strategies [48].

It is essential to proactively address sexual health concerns, irrespective of age. Screening for sexual symptoms during and after treatment for HM should be integrated into standard care and follow-up programs. Identifying sexual symptoms among older patients should lead to counselling or sexual rehabilitation programs. By integrating sexual health management at the stages of diagnosis, treatment, and follow-up care, HCPs can offer a more holistic approach to patient care, supporting patients throughout and following cancer treatment. This can potentially improve long-term outcomes and QoL as patients transition into survivorship [4, 10, 49].

This study is the first to investigate sexual health in older adults with HM and provides valuable insights into the sexual health of older adults with HM in Denmark. It highlights the prevalence of sexual symptoms and the factors that influence sexual activity and function. We conducted a sub-analysis of a larger cross-sectional study, which provides both strengths and limitations to the interpretation of our findings. A key strength of our study is the nationwide, diverse sample of older patients with HM, which enhances the representativeness and generalizability of our findings. However, a primary limitation is the inability to establish causal relationships due to the cross-sectional design, which cannot establish a temporal sequence between exposure and outcome variables. This is an explorative study and considering the small sample of subgroups the comparison of these should be interpreted with caution. One limitation of this study is the lack of multivariate analyses to adjust for potential confounders in the assessment of factors associated with sexual function. Statistical modeling was constrained by the unbalanced distribution of outcomes, separation issues caused by strong associations between some predictors and outcomes, and multicollinearity among patient-reported outcome measures. These challenges limited the stability and interpretability of multivariate models despite sufficient overall sample size. Consequently, results are presented as univariate associations, which may be subject to residual confounding. Future studies with larger or more balanced samples may enable more robust multivariate modeling to clarify these relationships. Sexual dysfunction may persist regardless of time since diagnosis which underscores the need for ongoing assessment and support for sexual health in patients with HM, including long-term survivors. Given the cross-sectional nature of this study, longitudinal research is essential to better understand individual trajectories of sexual recovery over time. Future research should incorporate prospective designs to clarify temporal patterns and inform tailored interventions that address the enduring sexual health needs of this patient population. Additionally, this sub-analysis is not based on a power calculation, and thus any temporal associations should be interpreted with caution. As this sub-analysis did not adjust for treatment type or intensity, we acknowledge that the findings should be interpreted with caution. The observed association may partly reflect underlying differences in disease burden or treatment side effects, rather than a direct effect of sexual activity on QoL. Our study did not include specific outcomes related to intimacy or the emotional aspects of sexual health. While general emotional functioning was assessed using the EORTC QLQ-C30, more nuanced measures of intimacy and emotional well-being were not available in our dataset. This represents an important area for future research to better capture the full scope of sexual health experiences in older adults with cancer. Moreover, cultural factors were not assessed, though it is important to note that the Danish population is relatively homogeneous in terms of cultural diversity. In both FSFI and IIEF, “not being sexually active” may reduce the overall score of sexual function. However, the original cross-sectional study analyzed sexual function specifically in a subgroup of sexually active individuals, with findings indicating that the level of dysfunction persists among those who were sexually active [22]. The clinical cut-off for sexual dysfunction is independent of age. Considering age-related changes in both men and women, the clinical cut-off for sexual dysfunction could potentially be lower than 25.66 and 26.0 in older adults, which is why the findings should be interpreted with caution. Finally, the evidence has pointed out that patients with less severe conditions are more likely to participate in research studies, which suggest a risk of the present study to underestimate the prevalence of sexual dysfunction in this population [50].

### Clinical implications

The findings have several important clinical implications. The association between sexual dysfunction and reduced QoL highlights the importance of addressing sexual health as a routine part of cancer care [22]. Our findings, along with existing evidence, suggest that sexual health concerns are relevant both during active treatment and in survivorship. Consistent with ASCO clinical guidelines, sexual health should be discussed proactively with patients from the time of diagnosis and revisited regularly throughout the care continuum [51]. Integrating sexual health into standard oncology care can help identify concerns early and ensure timely support, ultimately improving overall well-being and QoL for patients [52]. Given the high prevalence of sexual dysfunction in this population, HCPs should consider routinely screening for sexual symptoms. Early identification of sexual symptoms throughout the treatment trajectory is essential. Some patients may feel uncomfortable discussing sexual concerns, it is therefore, imperative to establish a supportive environment in which patients can address sexual symptoms openly. Educating HCPs about the potential risk of sexual dysfunction in older adults, particularly those with cancer, can help alleviate misunderstandings and reduce associated stigma. To provide holistic care that addresses the physical as well as psychosocial aspects of cancer treatment, stronger multidisciplinary collaboration between hematologists, nurses, psychologists, and sexual health specialists is required. Sexual health interventions for older adults with HM should consider several modifiable, age-related factors. These include physical comorbidities, medication side effects, fatigue, and psychosocial factors such as relationship dynamics, body image concerns, and emotional well-being. Emerging evidence supports the feasibility of both in-person and digital interventions such as tailored counseling, pelvic floor training, and web-based psychoeducational programs for addressing sexual dysfunction. For older adults, interventions may be enhanced by focusing on accessibility, partner involvement, and normalization of age-related changes in sexual function. Future studies should explore how to adapt existing sexual health interventions to meet the specific needs and preferences of older adults with cancer [53].

## Conclusions

In summary, this study indicates that older adults diagnosed with HM are at a significantly increased risk of experiencing poor sexual health. A high prevalence of sexual dysfunction was observed across all groups, regardless of diagnosis, age, and gender. However, men over the age of 75 exhibited more severe ED. Sexual dysfunction was accompanied by sexual distress, especially among men and patients with lymphoma. Patients with lymphoma reported more sexual symptoms, poorer QoL, and lower sexual activity, suggesting that this group is particularly vulnerable to sexual health challenges. Regardless of the treatment trajectory, older adults with HM experience sexual dysfunction. These findings underscore the need for consistent and supportive care addressing sexual health from diagnosis through treatment and into follow-up care.

## Data Availability

No datasets were generated or analysed during the current study.
